# Identification of *Toxoplasma gondii* Genes Responsive to the Host Immune Response during *In Vivo* Infection

**DOI:** 10.1371/journal.pone.0046621

**Published:** 2012-10-10

**Authors:** Sini Skariah, Dana G. Mordue

**Affiliations:** Department of Microbiology and Immunology, New York Medical College, Valhalla, New York, United States of America; Technion-Israel Institute of Technology, Israel

## Abstract

*Toxoplasma gondii* is an obligate intracellular protozoa parasite that causes the disease toxoplasmosis. It resides within host cells in a parasitophorous vacuole distinct from the host cell endocytic system. *T. gondii* was used as a model to investigate how obligate intracellular parasites alter their gene expression in response to the host immune response during infection compared to growth in host cells *in vitro*. While bacterial pathogens clearly alter gene expression to adapt to the host environment during infection, the degree to which the external environment affects gene expression by obligate intracellular pathogens sequestered within host cells is less clear. The global transcriptome of *T. gondii* was analyzed *in vivo* in the presence and absence of the IFN-γ-dependent host innate immune response. The parasites' *in vivo* transcriptome was also compared to its transcriptome *in vitro* in fibroblast cells. Our results indicate that the parasite transcriptome is significantly altered during *in vivo* infection in the presence, but not absence, of IFN–γ-dependent immunity compared with fibroblasts infected *in vitro*. Many of the parasite genes increased *in vivo* appear to be common to an early general stress response by the parasite; surprisingly putative oocyst stage specific genes were also disproportionately increased during infection.

## Introduction

Most bacterial pathogens significantly alter their transcriptome within a host during infection compared to growth *in vitro* in culture. To do this pathogens must be able to sense changes in their microenvironment and translate those signals into alterations in gene expression important for pathogenesis including but not limited to protection against the host immune response. The degree to which obligate intracellular pathogens must sense and respond to their environment is unknown; as it is unclear how the environment in a host cell *in vitro* differs to that encountered *in vivo* during infection and how the immune response of the host effects this environment. Changes in the global transcriptome of a pathogen can be used as a tool to interrogate the specific specialized intracellular niche occupied by a pathogen during *in vivo* infection and how the environment in this niche is altered by a progressive immune response [1].

*T. gondii* is an obligate intracellular protozoal pathogen. It actively invades virtually any type of warm blooded vertebrate host cell [2], independent of phagocytosis, and replicates within a parasitophorous vacuole (PV) that is largely segregated from the endocytic system [3], [4], [5]. This is markedly distinct from the majority of intracellular pathogens that require uptake by phagocytosis and formation of a nascent phagosome prior to pathogen mediated vacuole modification or escape from the phagosome into the cytosol.

A number of elegant global gene expression studies have analyzed changes in gene expression during the parasite's lytic cycle *in vitro* in human fibroblasts (HFF cells) [6], [7], [8]. These have shown that *T. gondii's* transcriptome is highly cell cycle regulated with many genes transcribed maximally just prior to their use and then downregulated in a pattern described as “just in time” [7], [8]. For example, genes important for parasite invasion of host cells are transcribed maximally just prior to parasite lysis from the host cell.

*T. gondii* also exhibits distinct developmental stages each with defined alterations in parasite gene expression. The asexual stages are the relatively quiescent bradyzoite stage contained within tissue cysts during chronic infection and the rapidly replicating tachyzoite stage that predominates during acute infection and reactivation of chronic infection. Feline hosts are the only known definitive host for the parasite where sexual reproduction occurs resulting in the fecal release of environmentally resistant oocysts that undergo sporulation in the environment to become infectious. Peroral infection with bradyzoite containing cysts or sporulated oocysts results in cyst rupture, invasion of the intestinal epithelium followed by differentiation to and systemic dissemination of the tachyzoite stage. A vigorous IFN-γ-dependent cell mediated immune response eventually controls the tachyzoite stage of infection. However, some tachyzoites undergo a developmental switch to bradyzoites contained within tissue cysts and these persist long term largely in muscle tissue and the CNS. These bradyzoites can re-activate back to tachyzoites causing severe pathology in individuals with a suppressed IFN-γ-dependent cell mediated immune response. Global gene expression studies in combination with previous EST date are beginning to elucidate transcriptomes unique to the tachyzoite, bradyzoite and oocyst stages of *T. gondii* development [9], [10], [11].

Although the *T. gondii* transcriptome is highly cell cycle regulated and the parasite differentiates between developmental stages, it is unclear how the parasite senses changes in its environment to trigger changes in its transcriptome. To date, two-component regulatory systems or other defined mechanisms for triggering transcriptional responses to extracellular environmental changes have not been described for *T. gondii*.

In this study we utilized global microarray expression analysis to identify the degree to which *T. gondii* alters its transcriptome in response to the *in vivo* microenvironment during the innate immune response (four days post-infection) relative to growth in human fibroblast (HFF) cells *in vitro*. To distinguish changes in the parasite transcriptome dependent or independent of the host immune response the parasite transcriptome was examined in IFN-γ gene deleted mice in addition to wild type (WT) mice. IFN-γ is the key cytokine required for inhibition of *T. gondii* replication *in vivo* and in its absence parasite growth is largely unrestrained [12]. Our results indicate that the parasite transcriptome is significantly altered during *in vivo* infection in the presence, but not absence, of IFN–γ-dependent immunity compared with fibroblasts infected *in vitro*. Therefore parasite transcriptional changes are largely induced in response to the IFN–γ-dependent-innate immune response. This also suggests that in the absence of host immunity the *in vitro* environment in HFF cells versus *in vivo* in the peritoneum is relatively similar from the parasites' perspective.

## Results

### Biological model used for parasite global transcriptome analysis

Whole-genome expression profiling was performed using the Affymetrix ToxoGeneChip microarray [13] to analyze the transcriptome response of *T.gondii* to the *in vivo* microenvironment during acute infection in the presence and absence of IFN-γ-dependent immunity. This array interrogates 8,058 predicted *T. gondii* genes (version 4.0 genome annotation) using 3′ biased probes. The parasite transcriptome was compared between tachyzoites grown *in vitro* in HFF cells to parasites isolated from the peritoneum of infected WT or IFN-γ KO mice on day four post-infection. Comparing the transcriptome of the parasite in both WT and IFN-γ KO mice allowed identification of parasite transcriptome changes induced in response to the host immune response versus those dependent on the *in vivo* microenvironment but independent of IFN-γ. The Type II genotype strain Prugniaud was utilized for these studies. The Type II genotype readily switches from tachyzoites to bradyzoites allowing analysis of tachyzoite to bradyzoite conversion during infection in the presence and absence of endogenous IFN-γ. The Type II genotype is also the most common cause of human infections [14].

**Figure 1:**
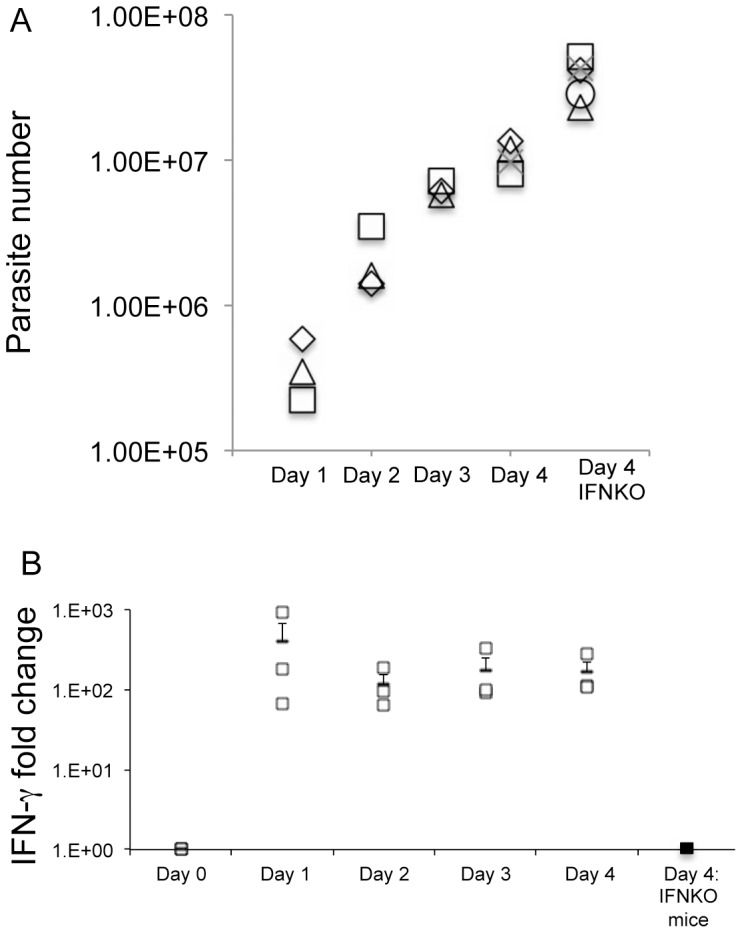
Kinetic analysis of parasite number and IFN-γ production in the peritoneum of infected mice. A. Parasite numbers increased in WT and IFN-γ KO mice throughout the first four days of infection. B. IFN-γ levels are increased in the peritoneum within 24 hours of infection. Values are for WT mice except for those specifically designated as IFNKO. Symbols represent individual mice. The mean and standard error were calculated from three to five mice for each day. IFN-γ production in WT mice was significantly increased on day 1–4 post infection compared to day 0 (p<0.05). Parasite numbers between WT and IFN-γ KO mice were statistically different at day four post infection (p<0.05). IFN-γ was undetectable in IFN-γ KO mice throughout infection.

For *T. gondii* global expression analysis mice were infected with 2×10^6^ tachyzoites IP and on day four post infection parasites were isolated from the peritoneum for microarray analysis. The number of viable parasites in the peritoneum was determined on days one through four post-infection in WT mice and on day four post-infection in IFN-γ KO mice by a modified plaque assay (see Materials and Methods). As shown in figure 1A numbers of parasites increased over the four days in WT mice although on day one post infection the number was decreased relative to input parasites. As expected parasite number on day four post infection was higher in IFN-γ KO mice relative to WT mice consistent with the requirement for IFN-γ in the control of *T. gondii*.

To determine the length of parasite exposure to endogenous IFN-γ during the four day infection, the kinetics of IFN-γproduction were evaluated by real time qRT-PCR. Sustained upregulation of IFN-γ occurred within the first day post-infection in WT mice and was absent as expected in the IFN-γ KO mice throughout infection (Fig. 1B). This indicated parasites in WT mice were exposed to increased levels of endogenous IFN-γ for at least three days prior to collection for microarray analysis.

Array analysis compared the transcriptome of parasites isolated from WT mice or IFN-γ KO mice relative to tachyzoites in HFF cells *in vitro* 48 hours post-invasion (approximately 85% intracellular). Parasites in HFF cells were chosen as a comparison for two reasons. First, HFF cells are the most common cell type used for studies on the parasite's lytic cycle as well as parasite transcriptome analysis allowing us to compare our *in vivo* results to the *in vitro* transcriptome studies in the field. Second, parasite replication in naïve murine macrophages appears largely unimpaired similar to that for HFF cells *in vitro* (data not shown).

### Endogenous IFN-γ exerts a pronounced effect on the *T. gondii* transcriptome during infection

**Figure 2:**
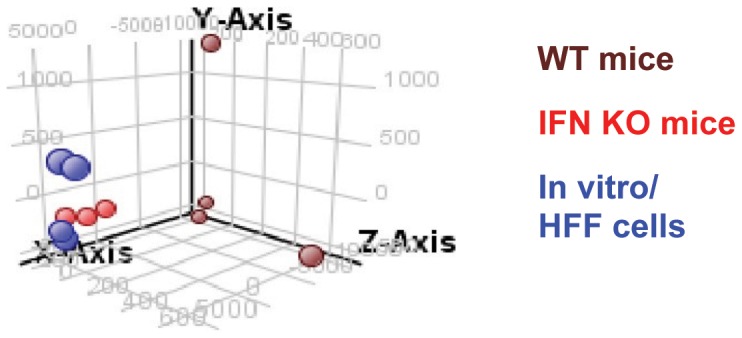
Genome wide expression analysis indicates a significant difference between parasites isolated from WT mice compared to those grown *in vitro* in HFF cells and *in vivo* in IFN KO mice. Principal component analysis of replicate samples of parasites grown *in vitro* in HFF cells compared to *in vivo* in WT mice or IFN KO mice. Principle component analysis (PCA) captures the variation across samples using a limited number of parameters to estimate sample to sample differences. In this study the first principle component (X-axis) captures 73.47 percent of the variance. Y and the Z axis show two minor trends which represent 5.38 and 2.8 percent variance respectively.

Principle component analysis (PCA) was performed on the microarray results from the independent *in vitro* and *in vivo* WT and IFN-γ KO parasite samples to examine the major trends in the data and distances between samples (Fig. 2). Both the *in vitro* parasites and the parasites isolated from IFN-γ KO mice displayed similar variance and were highly correlated with one another. In contrast, parasites isolated from WT mice with an intact IFN-γ-dependent immune response did not correlate with parasites *in vitro* or in IFN-γ KO mice and had significantly greater variance.

**Figure 3:**
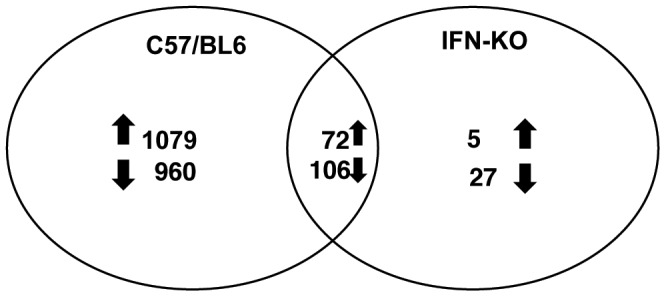
Venn diagram showing number of parasite genes with significant differences in expression *in vivo* in WT or IFNKO mice compared to *in vitro* in HFF cells. Diagram shows the number of parasite genes that were (↑) induced/(↓) repressed in wild type C57/BL6 and IFN-γ KO mice compared to *in vitro* in HFF cells. Genes showing ≥2 fold difference and p values <0.01 for comparisons between *in vitro* and *in vivo* (WT) and p<0.05 when comparing *in vitro* and *in vivo* (IFNKO) samples were deemed significant.

In figure 3, a Venn diagram shows the number of parasite genes increased or decreased in parasites isolated from WT or IFN-γ KO mice compared to *in vitro*. The criteria for designation of differential expression between *in vivo* WT and *in vitro* samples was a greater than 2-fold change and determined as significantly changed by both a) unpaired t-test with benjamini hochberg correction (p<0.01) and by b) SAM analysis [FDR, False Discovery Rate (90th percentile): 0.04939%, Delta value: 1.603]. The stringency was relaxed to p<0.05 for changes between *in vitro* and IFN-γKO. For all analysis in the paper genes designated as increased or decreased *in vivo* reflect at least a 2 fold change in addition to the above criteria for significance. Parasites isolated from infected WT mice had 1151 genes whose transcription was upregulated and 1066 that were downregulated compared to parasites grown *in vitro* in HFF cells. In contrast, only 72 of these gene transcripts were significantly upregulated and 106 downregulated in IFN-γKO mice compared to *in vitro*. There was a small subset of parasite gene transcripts that were altered only in IFN-γ KO mice and not in WT mice. Five of these were upregulated only in IFN-γ KO mice and 27 were downregulated. All the genes with differential expression between the various groups are listed in Tables S1 and S2. The observation that parasite changes in gene expression generally required the presence of IFN-γ to reach significance levels suggests that the *in vivo* environment the parasite encounters in the absence of IFN-γ relative to *in vitro* growth in HFF cells is not substantially different from the perspective of the parasite.

### Real time qRT-PCR validation of parasite genes representing select patterns of differential expression

Microarray analysis identified four broad categories of transcriptional alterations in *T. gondii in vivo* compared to *in vitro* in HFF cells. The largest set of parasite transcripts induced *in vivo*, 1079, required the presence of IFN-γ as they were only altered in WT mice and not in IFN-γ KO mice. These were designated *in vivo*, IFN-γ dependent. A smaller set of 72 transcripts was induced in parasites isolated from both WT and IFN-γ KO mice. These were designated IFN-γ independent-*in vivo*-induced transcripts. A very small number of genes (5) were upregulated *in vivo* but only in the absence of IFN-γ. Finally, a large number of parasite genes were downregulated in WT mice (960), WT and IFN-γ KO mice (106) or only IFN-γ KO mice (27) relative to *in vitro* grown tachyzoites. A subset of genes was selected from the above expression patterns for evaluation by qRT-PCR to validate the microarray results. Table 1 shows the qRT-PCR and microarray expression fold change of ten predicted *T. gondii* genes in parasites isolated from WT or IFN-γ KO mice relative to *in vitro* in HFF cells. The primers used for the PCR are listed in Table S3. Genes 44.m02656 (TGME49_031360), 59.m00026 (TGME49_069170), and 27.m00833 (TGME49_010950) were all preferentially upregulated in parasites isolated from WT mice relative to *in vitro*. TGME49_031360 is a hypothetical protein with a putative signal peptide and mass spectrometry data showing expression in both the conoid-enriched and conoid-depleted fractions in tachyzoites, after omitting proteins identified as contaminants derived from other subcellular organelles [15]. TGME49_069170 and TGME49_010950 are both hypothetical proteins with expression in partially sporulated oocysts based on EST and microarray data [10], [16]. 76.m03412 (TGME49_084310) and 113.m01577 (TGME49_097280) were induced preferentially in IFN-γ KO mice relative to *in vitro*. TGME49_084310 is a hypothetical protein and TGME49_097280 is a hypothetical protein with a putative prokaryotic membrane lipoprotein lipid attachment site. Both proteins have a putative signal peptide and show increased expression in bradyzoites and sporulated oocysts as opposed to tachyzoite stage [10], [16]. Mass spectrometry data also shows expression of TGME49_097280 in the oocyst stage [16]. 72.m00003 (TGME49_080580), 42.m03338 (TGME49_024170), 49.m03400 (TGME49_044260), 37.m00739 (TGME49_017400) and 31.m00871 (TGME49_013050) were all decreased *in vivo* in both WT and IFN-γ KO mice during infection compared to *in vitro*. TGME49_080580 is a classical bradyzoite differentiation antigen, P18 bradyzoite surface antigen/SAG4.2 [17]. TGME49_024170 is a putative SRS domain-containing protein common to a family of parasite GPI-anchored surface proteins some of which are developmentally expressed [18]. Microarray results for *in vitro* and *in vivo* generated bradyzoites imply enriched expression in bradyzoites for both genes [11]. The others are hypothetical proteins but proteomic data is available for TGME49_044260 and TGME49_013050 in the tachyzoite stage of the parasite.

**Table 1:** Fold change in gene expression by microarray versus qRT-PCR for in vivo conditions versus *in vitro* in HFF cells.

		Fold change WT/HFF		Fold change IFNKO/HFF	
Gene ID (ToxoDBv4)	Function	Array	qRTPCR	Array	qRTPCR
ME49_031360 (44.m02656)	Hypothetical	3.47	2.30(0.03)	1.53	1.53
ME49_069170 (59.m00026)	Hypothetical	4.15	4.07 (0.77)	1.65	2.04 (0.89)
ME49_010950 (27.m00833)	Hypothetical	8.51	3.60 (2.98)	1.91	2.13 (0.62)
ME49_084310 (76.m03412)	Hypothetical	1.10	4.7 (2.2)	6.05	12.13 (2.2)
ME49_097280 (113.m01577)	Hypothetical	0.90	2.20	2.4	6.14 (0.26)
ME49_080580 (72.m00003)	P18	−7.45	−10.5 (3.23)	−4.35	−5.02 (1.68)
ME49_024170 (42.m03338)	Hypothetical	−2.68	−2.50	−3.23	−3.85 (0.03)
ME49_044260 (49.m03400)	Hypothetical	−25.79	−7.25	−5.42	−6.66
ME49_017400 (37.m00739)	Hypothetical	−5.46	−6.67	−7.87	−10.43 (0.29)
ME49_013050 (31.m00871)	Hypothetical	−14.05	−11.11	−17.32	−14.02 (1.54)

Parenthesis indicate standard deviation between multiple biological replicates.

### *T gondii in vivo* transcriptome does not correlate to a specific parasite cell cycle stage

IFN-γ is important for activating host cells to restrict growth of *T. gondii* and for host cell cytolytic activity against the parasite. In the absence of IFN-γ parasite replication is relatively unrestrained. Approximately 40% of *T. gondii* genes have a significant cell cycle expression pattern in the tachyzoite stage [8]. To investigate the potential contribution of parasite cell cycle inhibition by innate immunity to the *in vivo* transcriptome, the parasite genes induced *in vivo* were compared to those previously shown to be cell cycle regulated [8]. Only 11.8% of the 1151 genes increased *in vivo* were designated as cell cycle regulated genes (137 total). These are highlighted in Table S2. 62 of these were classified as G1 and 75 as S/M phase. This suggests that the *T. gondii in vivo* transcriptome is not simply a consequence of parasite cell cycle arrest by IFN-γ-dependent innate immunity.

### Transcripts for parasite stress response genes are induced early during acute infection in the presence of IFN-γ-dependent innate immunity

Although tachyzoite to bradyzoite conversion can be induced within a few days [11], [19], [20], the degree and kinetics of conversion *in vivo* during acute infection in the peritoneum and lymphoid tissue is less clear. To examine the extent to which bradyzoite specific gene transcription was induced four days post-infection in the peritoneum, the array expression values of a panel of known bradyzoite genes were examined from parasites isolated from WT mice compared to HFF cells *in vitro*. These included bradyzoite antigen-1 (BAG1), ENO1, bradyzoite rhoptry protein 1(BRP1), LDH2, SAG2Yand SRS 9 [11], [21]. Transcripts of tachyzoite-specific genes, including ENO2, LDH1, SAG1, SAG2A and SRS2 were also examined [11], [21]. Results are shown in Table 2. None of the canonical bradyzoite genes were significantly increased *in vivo* during infection based on our statistical cut offs. Expression of two parasite bradyzoite genes TGME49_080580 (P18/SAG 4.2) and TGME49_024170 (SRS-domain-containing protein) were further evaluated by qRT-PCR; both were downregulated in parasites from WT and IFN-γ mice relative to *in vitro* (Table 1). Our microarray results were also compared with genes induced by compound 1, a trisubstituted pyrrole that was identified in a small molecule screen as a potent inducer of bradyzoite-specific antigens [22]. Out of the 94 parasite genes altered by compound 1 *in vitro*, only 13 were statistically increased *in vivo* relative to *in vitro* in the current study (Table S4). These results in total indicate that at least at day four post-infection tachyzoite to bradyzoite conversion is not strongly triggered in the peritoneum by the host innate immune response.

**Table 2:** Microarray fold change of canonical bradyzoite genes during infection in WT or IFNKO mice compared to HFF cells *in vitro*.

Gene ID (ToxoDBv4)	Name	Function	Fold change WT/HFF	Fold change IFNKO/HFF
*Genes known to have increased expression in bradyzoites compared to tachyzoites*				
ME49_05920 (55.m00009)	BAG1/HSP30	Heat shock protein	1.35	1.16
ME49_068860 (59.m03411)	ENO1	Enolase 1	1.97	1.27
ME49_114250 (583.m09133)	BRP1	Bradyzoite rhoptry protein	−1.06	4.43
ME49_007130 (23.m00149)	SAG2Y/SRS49A	Surface antigen	1.91	−1.08
ME49_120190 (641.m01562)	SRS9/SRS16B	Surface antigen	1.63	1.02
ME49_091040 (80.m00010)	LDH2	Lactate dehydrogenase 2	2.59	1.13
*Genes known to have decreased expression in bradyzoites compared to tachyzoites*				
ME49_068850 (59.m03410)	ENO2	Enolase 2	1.02	1.28
ME49_071050 (59.m00008)	SAG2A/SRS34A/P22	Surface antigen	1.06	1.03
ME49_033480 (44.m00010)	SRS2/p35	Surface antigen	1.06	1.03
ME49_033460 (44.m00009)	SAG1/SRS29B/P30	Surface antigen	1.39	1.12
ME49_032350 (44.m00006)	LDH1	Lactate dehydrogenase 1	−1.62	−1.45

Although mature bradyzoite transcripts were not significantly induced during infection, it was possible that early stress response genes were induced during infection in the presence of IFN-γ-dependent innate immunity. To examine this possibility the current data set was compared with data sets for parasite genes induced by sodium nitroprusside (SNP), a nitric oxide donor, or by CO_2_ starvation (ToxoDB.org; Bradyzoite Differentiation (Multiple 6-hr time points and Extended time series; Paul Davis, Florence Dzierszinski and David Roos). The Bradyzoite Differentiation Study identified 909 parasite transcripts induced greater than 2-fold 24 hours after SNP treatment. Approximately 45% of these transcripts were also statistically increased *in vivo* in the presence of an intact immune response in the current study. These genes are listed in Table S4. In comparison, 211 transcripts were identified in the Bradyzoite Differentiation Study as upregulated greater than 2 fold in CO_2_ depleted conditions for 30 hours. 33% of these transcripts were also statistically increased *in vivo* in parasites isolated from WT mice (Table S4). These results suggest that the *in vivo* microenvironment in the presence of an intact IFN-γ dependent innate immune response induces alterations in parasite gene expression consistent, in part, with an early general stress response induced *in vitro* by SNP or CO_2_ depletion.

### Transcription of parasite genes coding for putative surface proteins and intracellular signaling cascades are disproportionately increased *in vivo* in the presence of IFN-γ

**Figure 4:**
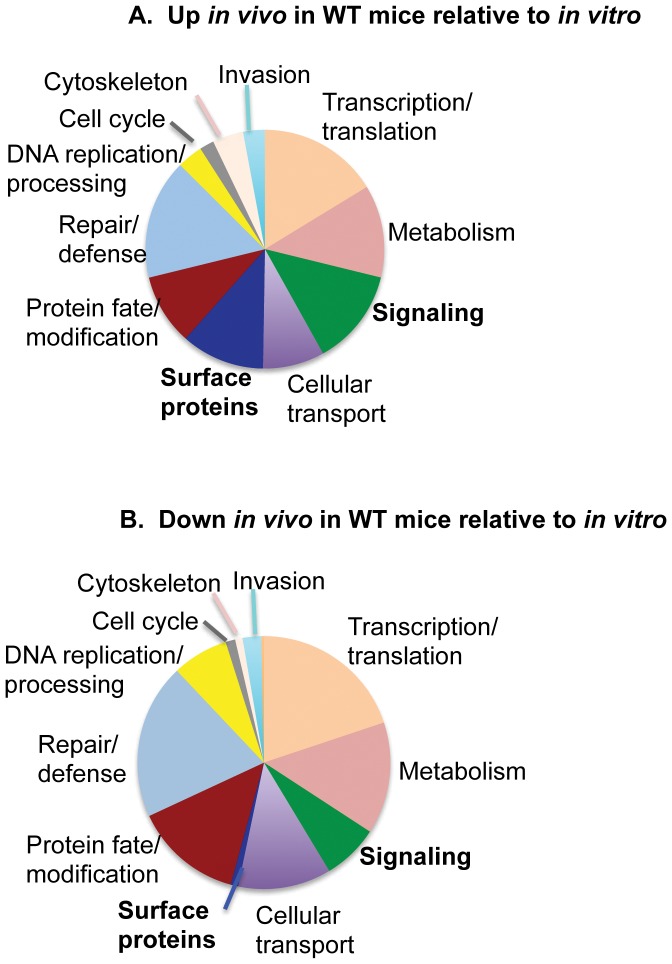
Functional categorization of *T. gondii* genes with increased or decreased expression in WT mice compared to *in vitro* in HFF cells. A. Functional categorization of genes increased *in vivo* in WT mice compared to *in vitro*, B. Functional categorization of genes decreased *in vivo* in WT mice compared to *in vitro.* 68% of the genes up in WT mice and 51% of the genes down in WT mice are of unknown function and are not included.

Genes statistically upregulated *in vivo* during infection relative to *in vitro* in HFF cells were classified into 11 categories based on known or implied function. The data are displayed as a pie chart with slices representing the proportion of genes in each category. Genes of unknown function are not included in the pie chart but comprised the majority of the genes. 68% of the genes up in parasites from WT mice and 51% of the genes down in parasites from WT mice were of unknown function. Figure 4A shows the functional categorization of *T. gondii* genes upregulated *in vivo* in WT mice compared to *in vitro*. This is compared to Figure 4B that shows categorization of genes downregulated *in vivo* in WT mice compared to *in vitro*. Two categories in particular appeared increased *in vivo* compared to those downregulated *in vivo*. Genes implicated in intracellular signaling were increased slightly preferentially *in vivo*. Putative surface proteins, the majority with putative prokaryotic membrane lipoprotein lipid attachment sites as evaluated by ToxoDB (ProSite), were also preferentially upregulated *in vivo* compared to the proportion downregulated *in vivo*. Prokaryotic membrane lipoprotein attachment site profiles for proteins are based on the presence of a putative defined signal peptide that is cleaved by a specific signal peptidase II upstream of a cysteine residue to which a glyceride-fatty acid lipid is attached. The putative surface proteins did not include transmembrane proteins characterized as putative transporters that were instead included in the category cellular transport.

### Transcripts for oocyst genes are disproportionately increased *in vivo* during infection

**Figure 5:**
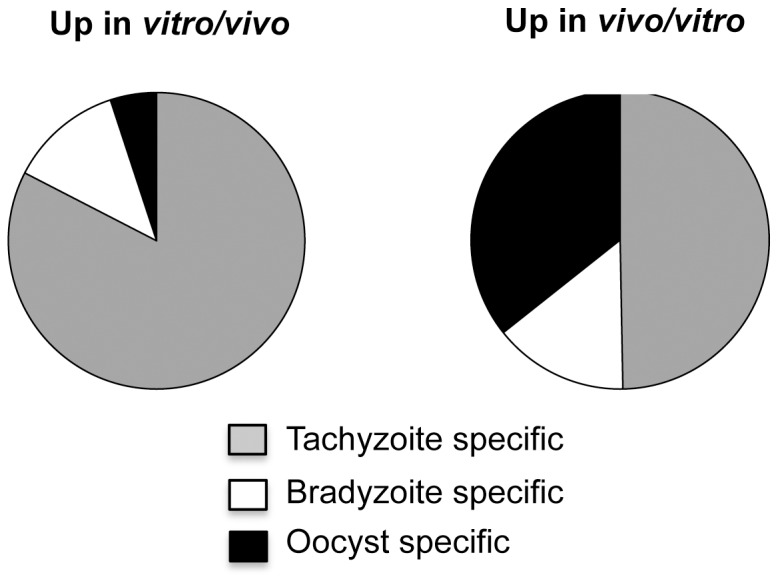
A disproportionate number of putative oocyst stage specific genes are increased *in vivo* in WT mice compared to *in vitro* in HFF cells. *T. gondii* tachyzoite, bradyzoite and oocyst specific transcripts that were differentially expressed by *T. gondii in vivo* in WT mice versus *in vitro* in HFF cells For the comparison, stage specific genes were those that had EST data for only one stage of the parasite and/or at least a 10-fold preferential increase in the ToxoDB microarray data for a particular stage.

To more broadly examine the effect of *in vivo* infection and the host innate immune response on parasite stage specific gene expression, genes with increased and decreased expression *in vivo* in WT mice were interrogated using data on ToxoDB (http://ToxoDB.org) for stage specific ESTs or transcripts for either tachyzoites, bradyzoites or oocysts. For the comparison, stage specific genes were those who had EST data for only one stage of the parasite and/or at least a 10-fold preferential increase in the ToxoDB microarray data for a particular stage. Figure 5 shows the distribution of tachyzoite, bradyzoite and oocyst specific transcripts that were differentially expressed by *T. gondii in vivo* in WT mice versus *in vitro* based on these criteria. Out of the 171 stage specific genes increased in parasites from WT mice, 85 of the genes were tachyzoite specific, 25 bradyzoite specific and 61 oocyst specific (including partially and fully sporulated oocysts). The bradyzoite and oocyst specific genes increased *in vivo* in WT mice are listed in Table 3. qRT-PCR confirmed *in vivo* induction of two of the oocyst genes, TGME49_069170 and TGME49_010950 (Table 1). In the absence of IFN-γ, only three oocyst specific transcripts were increased *in vivo* suggesting that host immunity was important for the increased expression of the oocyst-associated genes.

**Table 3:** Bradyzoite and oocyst specific genes increased in WT or IFNKO mice.

Gene ID	ToxoDBv4	Function	Fold change WT/HFF	Stage (EST)
*ME49_018490*	*38.m01047*	*Hypothetical*	*6.77*	*Tachy subtract.*
ME49_003210	20.m05971	Hypothetical	4.59	Bradyzoite
ME49_052640	52.m01553	Membrane ATPase	4.45	Bradyzoite
ME49_070580	59.m03253	Ubiquitin ligase	4.14	Tachy subtract.
*ME49_114860*	*583.m05644*	*Zinc knuckle-domain*	*3.95*	*Tachy subtract.*
ME49_044050	49.m03386	Hypothetical	3.81	Tachy subtract.
ME49_028480	42.m07433	Hypothetical	3.78	Bradyzoite
ME49_073940	59.m06081	Hypothetical	3.77	Bradyzoite
ME49_030900	44.n02622	Hypothetical	3.65	Tachy substract.
ME49_031360	44.m02656	Hypothetical	3.47	Tachy subtract.
ME49_009750	25.m01887	Hypothetical	3.33	Bradyzoite
ME49_036970	46.m01723	SNF-domain	3.09	Bradyzoite
ME49_074030	59.m03719	Hypothetical	3.09	Bradyzoite
ME49_113490	583.m05560	Hypothetical	2.79	Bradyzoite
ME49_099090	129.m00514	Hypothetical	2.60	Bradyzoite
ME49_021710	41.m01320	TBC domain	2.55	Bradyzoite
ME49_050800	50.m03372	Hypothetical	2.54	Bradyzoite
ME49_008020	25.m01794	Hypothetical	2.42	Bradyzoite
ME49_101790	173.m00003	Hypothetical	2.42	Bradyzoite
ME49_033160	44.m02771	Hypothetical	2.36	Tachy subtract.
ME49_069630	59.m03461	Hypothetical	2.22	Tachy subtract.
ME49_019780	38.m02375	Hypothetical	2.10	Bradyzoite
ME49_090690	80.m05034	Hypothetical	2.09	Bradyzoite
ME49_015300	33.m01356	Hypothetical	2.08	Bradyzoite
*ME49_010770*	*27.m00073*	*Hypothetical*	*9.13*	*Partially sporulated*
*ME49_076860*	*65.m01087*	*Late embryogenesis domains*	*8.64*	*Fully sporulated*
ME49_002110	20.m03695	Hypothetical	7.09	Partially sporulated
ME49_119680	641.m01531	Hypothetical	6.98	Partially sporulated
ME49_033210	44.m02776	Hypothetical	5.61	Oocyst
ME49_116560	583.m05760	Hypothetical	5.39	Partially sporulated
ME49_066130	57.m03094	Peroxidase putative	5.19	Partially sporulated
ME49_014570	33.m01293	Hypothetical	5.17	Partially sporulated
ME49_042390	49.m00036	Enoyl CoA hydratase	4.55	Partially sporulated
ME49_069110	59.m00004	Ornithine amino trans.	4.49	Partially sporulated
ME49_030540	44.m00025	Hypothetical	4.23	Partially sporulated
ME49_069170	59.m00026	Hypothetical	4.15	Partially sporulated
ME49_115090	583.m05654	Hypothetical	4.11	Partially sporulated
ME49_025460	42.m00040	Hypothetical	4.10	Partially sporulated
ME49_111420	583.m09193	Hypothetical	3.99	Partially sporulated
ME49_116290	583.m00700	Hypothetical	3.94	Partially sporulated
ME49_019220	38.m01104	Hypothetical	3.85	Partially sporulated
ME49_008770	25.m02933	Hypothetical	3.77	Partially sporulated
ME49_113000	583.m00677	Hypothetical	3.69	Partially sporulated
*ME49_061560*	*55.m00160*	*TBPIP domain*	*3.65*	*Partially sporulated*
ME49_078620	65.m011181	Hypothetical	3.51	Partially sporulated
ME49_078690	65.m01958	Hypothetical	3.48	Partially sporulated
ME49_030530	44.m04676	Hypothetical	3.45	Partially sporulated
ME49_067410	57.m01855	Scavenger Receptor	3.36	Partially sporulated
ME49_037080	46.m01732	Hypothetical	3.29	Partially sporulated
ME49_088310	80.m00037	Hypothetical	3.13	Partially sporulated
ME49_028440	42.m07382	Hypothetical	3.12	Partially sporulated
ME49_002090	20.m03693	Hypothetical	3.10	Partially sporulated
ME49_048760	50.m03259	Serine protease	2.99	Partially sporulated
ME49_062430	55.m04989	Synthase	2.97	Fully sporulated
ME49_078570	65.m00025	Hypothetical	2.96	Partially sporulated
ME49_078110	65.m01151	1,3B-glucan synthase	2.87	Partially sporulated
ME49_104450	540.m00325	Hypothetical	2.82	Partially sporulated
ME49_017380	37.m00738	Hypothetical	2.82	Partially sporulated
ME49_032470	44.m04635	Hypothetical	2.77	Partially sporulated
ME49_115350	583.m05674	Hypothetical	2.76	Partially sporulated
ME49_120540	641.m01583	Hypothetical	2.74	Partially sporulated
ME49_059190	55.m00114	Cysteine synthase	2.72	Partially sporulated
ME49_024810	42.m03380	Hypothetical	2.70	Partially sporulated
ME49_109320	583.m05285	SRS domain	2.67	Partially sporulated
ME49_093240	83.m01199	Hypothetical	2.64	Partially sporulated
ME49_080430	72.m00381	Hypothetical	2.62	Partially sporulated
ME49_109540	583.m05292	Hypothetical	2.62	Partially sporulated
ME49_105280	541.m01167	Hypothetical	2.57	Partially sporulated
ME49_085790	76.m01619	Hypothetical	2.56	Partially sporulated
ME49_023700	42.m03301	LCCL-domain	2.55	Partially sporulated
ME49_085690	76.m01612	Notch DSL-domain	2.54	Partially sporulated
ME49_057460	55.m00066	Hypothetical	2.52	Partially sporulated
ME49_053110	52.m01565	Kinesin motor-domain	2.50	Partially sporulated
ME49_069280	59.m06119	Hypothetical	2.44	Partially sporulated
ME49_002100	20.m03694	Hypothetical	2.43	Partially sporulated
ME49_054850	52.m02707	Hypothetical	2.39	Partially sporulated
ME49_072510	59.m06075	Aspartyl protease	2.39	Partially sporulated
ME49_011840	28.m00282	Hypothetical	2.39	Partially sporulated
ME49_094600	83.m00008	Hypothetical	2.34	Partially sporulated
ME49_026100	42.m03480	Copper-transporter	2.21	Partially sporulated
ME49_034570	46.m01618	Peroxisomal enzyme	2.21	Partially sporulated
ME49_042590	49.m03294	Hypothetical	2.18	Partially sporulated
ME49_066770	57.m00035	CPW-WPC domain	2.17	Partially sporulated
ME49_039660	49.m03163	Hypothetical	2.13	Partially sporulated
ME49_008330	25.m00190	Hypothetical	2.12	Partially sporulated

Italics designate genes with increased expression in both WT and IFNKO mice relative to HFF.

## Discussion

This study represents the first global transcriptional analysis of how *Toxoplasma gondii*, an obligate intracellular protozoa, alters its transcription when it transitions from an intracellular environment *in vitro* in host cells to an *in vivo* environment in host cells during infection in the presence and absence of IFN-γ-dependent innate immunity. The study shows that the *in vivo* microenvironment in the absence of an IFN-γ-dependent innate immune response results in little transcriptional adaptation on the part of the parasite. This is consistent with the relative lack of restraint on parasite replication in HFF cells and murine macrophages *in vitro* and *in vivo* in the absence of IFN-γ. However, our results indicate the parasite clearly does respond to changes in the *in vivo* microenvironment due to the host IFN-γ-dependent innate immune response even early in infection. This response shares similarities with transcriptional changes induced *in vitro* by exposure to sodium nitroprusside (SNP), a nitric oxide donor or CO_2_ depletion.

It is clear from the present study that *T. gondii* senses its external environment from within its segregated compartment within a host cell and responds with alterations in gene expression in response to host immunity. It is well established that extracellular stresses can induce intracellular *T. gondii* to differentiate from tachyzoites to bradyzoites. However, this is the first study evaluating how, and the degree to which, the parasite responds transcriptionally to changes in the *in vivo* microenvironment during infection. The importance of these changes to parasite pathogenesis requires further investigation. How the parasite senses its environment is unknown. Our results suggest that the *in vivo* transcriptome is not simply a passive consequence on the part of the parasite due to an immune-induced parasite cell cycle block or inhibition of replication. To date, two-component regulatory systems used by bacteria to sense and respond to its environment have not been described for *T. gondii*. However, *T. gondii* could utilize proteins with redox-responsive residues such as iron-containing transcription factors to directly sense changes in redox potential to induce changes in gene expression similar to other pathogens [23], [24]. *Entamoeba histolytica* has an endoplasmic reticulum stress-sensing mechanism activated by nitric oxide important for modulating parasite nuclear gene expression [25]. Generation of second messengers such as cAMP downstream of phosphodiesterases mediates transduction of extracellular stimuli, such as nutrient deprivation, to intracellular downstream singling pathways in pathogenic fungi [26], [27]. In fact, Cyclic AMP and cGMP kinases have been implicated in *T. gondii* tachyzoite to bradyzoite conversion although the upstream signals remain unknown [28], [29].

The IFN-γ-dependent *T. gondii in vivo* transcriptome shares similarity with transcriptional changes induced *in vitro* in the first 24–30 hours of exposure to sodium nitroprusside (SNP) or low CO_2_. Both SNP and low CO_2_ can induce bradyzoite conversion *in vitro* during prolonged exposure. SNP is a nitric oxide donor with additional toxicity particularly to the mitochondria due to the release of cyanide molecules. IFN-γ can stimulate transcription of inducible nitric oxide synthase particularly in phagocytic cells resulting in nitric oxide that has cytostatic activity against *T. gondii*. Our results show that approximately 45% of the parasite genes induced *in vivo* overlapped with those previously identified as induced by SNP *in vitro* within 24 hours. This is likely due to the fact that nitric oxide is produced early during *T. gondii* infection of WT mice and may be mediating many of the *in vivo* IFN-γ-dependent transcriptional changes. However, approximately 30% of the genes induced *in vivo* overlapped with those induced *in vivo* following CO_2_ depletion. These common genes suggest that the parasite *in vivo* IFN-γ-dependent transcriptome response, in part, is indicative of a general early stress response in the parasite. This general stress transcriptional response may or may not be directly linked to eventual progression to mature bradyzoites.

In contrast to the overlap of genes of the IFN-γ-dependent *in vivo* transcriptome with genes up-regulated early after SNP addition or CO2 depletion, canonical bradyzoite markers were not increased and were sometimes decreased *in vivo* during infection. The fact that we did not observe progression to mature bradyzoite transcripts *in vivo* may be due to a four day or less exposure of parasites to an effective innate immune response. We show that IFN-γ is significantly induced within a day of parasite challenge. Although three days is sufficient to stimulate tachyzoite to bradyzoite conversion in terms of changes in gene expression *in vitro* with exogenous stress, it may not be long enough *in vivo*. However, it is also likely that tachyzoite to bradyzoite conversion occurs more readily in anatomical locations such as muscle and CNS rather than in primary lymphoid tissue where parasite clearance rather than bradyzoite conversion may be more likely.

Surprisingly a disproportionate number of the transcripts induced by the parasite during infection in an immune intact host are those traditionally considered as oocyst transcripts. Expressed sequence tags (EST) and global transcriptome analysis has largely been used to analyze gene expression for tachyzoites *in vitro*, bradyzoites or oocysts. This is the first study that evaluated the *T. gondii* tachyzoite transcriptome response to the *in vivo* microenvironment and innate immunity relative to *in vitro* culture in HFF cells. Consequently, some transcripts upregulated in oocysts may also be upregulated by *in vivo* factors during infection including host immunity.

A number of the transcripts increased during infection had low expression in tachyzoites grown in HFF *in vitro*. Transcripts with low expression can often be disproportionately represented in microarray analysis as a fold increase is more likely to be obtained for low expression transcripts. However, bradyzoite transcripts expected to be low or non-detectable in tachyzoites *in vitro* were not disproportionately increased *in vivo*. Therefore, the selective increase in a number of oocyst transcripts *in vivo* is unlikely to be explained strictly on the basis of their low expression *in vitro*. Similarly it is likely that parasite genes without evidence of gene transcription *in vitro* or in oocysts or bradyzoites may still be expressed *in vivo* during infection. The current study highlights the importance of evaluating gene expression during infection in addition to particular stages of parasite development before concluding a gene transcript is not expressed.

The majority of the parasite transcripts increased during infection was not annotated and lacked sufficient homology to other proteins to delineate potential functions. This is true for the parasite genome as a whole but is more pronounced with the genes whose transcripts are increased during infection. However, the increased expression of surface proteins with putative lipoprotein anchors *in vivo* relative to *in vitro*, suggests the possibility that up-regulation of these surface proteins may play a protective role in shielding the parasite. The other subset of proteins increased *in vivo* during infection were those involved in intracellular signaling consistent with the parasite responding to changes in its environment induced by the innate immune response.

This study establishes that *T. gondii's* transcriptome is responsive to the *in vivo* microenvironment early during infection in the presence of the IFN-γ-dependent innate immune response. In contrast, in the absence of IFN-γ, the parasite's transcriptome is surprisingly similar to that during growth *in vitro* in HFF cells. The *in vivo* transcriptome response shares similarities with what appears to be a general early stress response on the part of the parasite in the absence of bradyzoite conversion at least within the first four days of infection in the peritoneum. The unknown questions are how the parasite senses its environment to induce alterations in gene expression and the importance of such transcriptional adaptation early during infection.

## Materials and Methods

### Cell culture and parasite strains

The Prugniaud (Pru) strain of *T. gondii* (a gracious gift from Dr. Laura Knoll) was used in the microarray analysis and qRTPCR. Parasites were maintained in monolayers of human foreskin fibroblast cells (HFF) (ATCC; #CRL-2522) under standard *T. gondii* culture conditions [3]. HFF cells were grown to confluency in D10 media [Dulbecco's modified eagle's high glucose medium (DMEM) (HyClone, Logan, Utah), supplemented with 10% heat-inactivated fetal bovine serum (HyClone), L-Glutamine, 20 mM (HyClone), 1 unit/ml Penicillin and 1 unit/ml Streptomycin (HyClone)] at 37°C in a humidified 5% CO_2_ environment.

For *in vitro* microarray studies healthy confluent HFF cells were infected with *T. gondii*. A MOI was chosen to ensure the majority of HFF cells were infected and had one to two parasite-containing vacuoles (PVs). The cells were harvested 48 hours later as parasites were beginning to egress (10–30% extracellular). For *in vivo* transcriptome analysis C57BL/6 wild type (WT) or IFN gamma-gene deleted mice (IFNKO) were inoculated with 2×10^6^ parasites intraperitoneally (ip). Parasites were isolated from the peritoneal cavity of the mice four days post infection as previously described [30]. To ensure sufficient parasite RNA, parasites isolated from 3–5 mice were pooled together to serve as one biological replicate. Three to five biological replicates were analyzed per experimental group. To reduce host cell contamination, harvested samples were passed through a syringe and needle to rupture host cells and centrifuged to obtain a *T. gondii* pellet. Although this method reduced host cell contamination it was unable to effectively separate parasites from peritoneal cells resulting in significant host cell RNA in addition to parasite RNA. All animal experiments were conducted with the approval and oversight of the Institutional Animal Care and Use Committee at New York Medical College.

### RNA isolation, labeling, and microarray hybridization

Total RNA was isolated using RNeasy mini elute columns (Qiagen, Valencia). RNase inhibitor (Ambion, Inc) was added to samples followed by storage at −80°C. Synthesis, fragmentation of cRNA probes and hybridization of the samples to the *T. gondii* custom chip [13] by Affymetrix was performed by the University of Pennsylvania Microarray Facility (U.Penn) as per GeneChip Expression Analysis Technical Manual (Affymetrix, Santa Clara, CA).

### Preprocessing

Affymetrix Microarray suite 5.0 was used to quantify expression levels. Raw (.cel) files and the .cdf file were imported into RMADataConv in RMAExpress (http://rmaexpress.bmbolstad.com) [31], [32], [33]. A modified .cdf file was generated which excluded human and mouse genes present in the chip to eliminate any bias arising due to differences in the expression levels of host genes in the samples being compared during normalization. The output files containing only parasite genes were imported into RMAExpress. Gene expression values were quantified following normalization using Robust Multichip Average algorithm [31], [32], [33].

### Statistical analysis

Normalized expression data was analyzed using GeneSpring GX version 9 GX9 (Agilent Technologies, Inc. software. CA). Statistically significant differences in gene expression between experimental groups were determined using unpaired t-test with Benjamini Hochberg FDR correction to minimize false discovery. Genes showing ≥2 fold difference and p values <0.01 for comparisons between *in vitro* and *in vivo* (wt) and p<0.05 when comparing *in vitro* and *in vivo* (IFNKO) samples were selected.

Normalized data was also analyzed using two class unpaired SAM (significance analysis of microarrays) [34] as provided in MeV, multi experiment viewer version 4.3 [35], [36]. Fold differences ≥2 fold and a FDR, False Discovery Rate (90th percentile): 0.04939%, Delta value: 1.603 for comparisons between *in vitro* and *in vivo* (WT) and a FDR False Discovery Rate (90th percentile): 1.91679%, Delta value: 1.847 when comparing *in vitro* and *in vivo* (IFNKO) samples were set as parameters to select for differentially expressed genes. For the purpose of the present study, gene expression was only considered changed if a gene was changed >2 fold and considered significantly altered when analyzed by both SAM analysis and GeneSpring GX9. ToxoDB.org version 4.3 was initially used as a reference for identification of putative genes and data from later versions of ToxoDB (http://ToxoDB.org) up until version 7.1 was used for characterization of genes and proteins based on bioinformatics, information for functional characterization, ESTs and mass spectrometry and for many of the comparisons to published and pre publication microarray data.

### Real Time Quantitative RT-PCR

RNA samples were reverse transcribed with Superscript III (Invitrogen). cDNA was amplified using the FastStart Universal SYBR Green Master (ROX) (Roche Diagnostics) for regular assays and using FastStart Universal Probe Master (Roche Diagnostics) when using TaqMan® Gene Expression Assays (Applied Biosystems) on an ABI-7900 PCR system following the manufacturer's instructions (Applied Biosystems). Reactions containing cDNA generated without reverse transcriptase or containing primers alone were included as controls. Primers and probes used to amplify the genes are listed in Table S3. One to three biological replicates were analyzed in duplicate in this study. The cycle threshold (Ct) value was determined from a representative experiment unless specified otherwise. A relative quantitation method Delta Delta Ct [37] was used to determine the relative expression of a gene after normalization to housekeeping gene *T. gondii* actin. All of the probes utilized were tested against cDNA from uninfected mice to confirm specificity for parasites.

IFN-γ levels were determined using Taqman® Gene Expression Assay: Mm00801778_m1 after normalization to mouse glyceraldehyde-3-phosphate dehydrogenase with Taqman® Gene Expression Assay: Mm99999915_g1. The cycle threshold (Ct) value was determined from three biological replicates analyzed in triplicate. An unpaired Student's t-test was used to determine significant differences and p-values less than 0.05 were considered significant.

Parasites were also isolated from the peritoneal cavity of infected mice on days one through four post-infection to assess parasite viability by a modified plaque assay. Parasites were serial diluted and allowed to invade HFF cells in 96-well plates overnight. Cells were fixed with 2.5% formaldehyde buffered in PBS and stained with a rabbit polyclonal anti-*Toxoplasma* antibody (Fitzgerald Laboratories) followed by an Alexa488-conjugated goat anti-rabbit secondary antibody (Invitrogen). The number of PVs that contained more than 2 parasites per PV was counted in each well and that number was multiplied by the total dilution factor to determine the number of viable parasites in the peritoneal cavity of infected mice. Since we were unable to completely separate lymphoid cells from intracellular parasites, this method proved more accurate for quantification of the number of parasites in an infected cell than a traditional plaque assay.

### Microarray data accession number

The complete data set obtained in this study has been deposited in the ArrayExpress database E-MEXP-3579 “In vivo transcriptome analysis of *Toxoplasma gondii*”.

## Supporting Information

Table S1 **Genes whose expression is downregulated** ***in vivo*** **in wild type C57/BL6 and IFNKO mice compared to** ***in vitro*** **samples.**
(XLS)

Table S2 **Genes whose expression is increased** ***in vivo*** **in wild type mice and IFNKO mice compared to** ***in vitro*** **samples.** Highlighted rows show genes that are cell cycle regulated. Sheet 1, lists genes regulated *in vivo* in wild type mice only. Sheet 2, lists genes regulated *in vivo* in both wild type and IFNKO mice. Sheet 3, lists genes regulated *in vivo* in IFNKO mice only. Column descriptions: columns *in vitro* 1–4 show the normalized log expression values for genes in the individual *in vitro* samples. Columns *in vivo* 1–4 and IFNKO 1–3 show the normalized log expression values for genes in the individual *in vivo* samples from wild type mice and IFNKO mice respectively.
(XLS)

Table S3 **Primers and probes used to amplify genes.**
(XLS)

Table S4 **Genes whose expression is increased** ***in vivo*** **in wild type mice vs** ***in vitro*** **samples also induced by Compound 1, sodium nitroprusside (SNP) and reduced CO_2_.**
(XLS)
